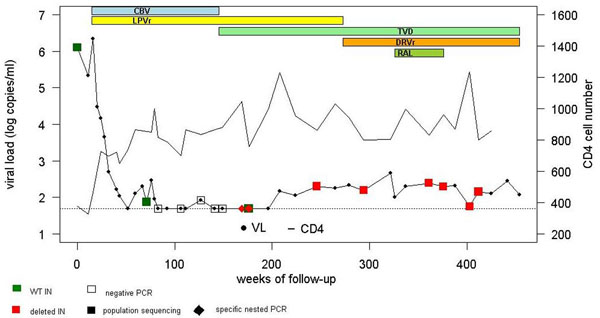# Persistent production of an integrase-deleted HIV-1 variant with no resistance mutation and wild-type proviral DNA in a treated patient

**DOI:** 10.1186/1471-2334-14-S2-O12

**Published:** 2014-05-23

**Authors:** MA Trabaud, L Cotte, J Saison, C Ramiere, C Ronfort, JC Tardy, P Andre

**Affiliations:** 1 Hospices Civils of Lyon, Lyon, France

## Introduction

An HIV-1-infected patient with suppressed viremia for several years, in whom a variant carrying a deleted integrase (IN) gene, without reverse transcriptase (RT) or protease (PR) resistance mutations, emerged in the plasma and persisted is described.

## Materials and methods

Viral load (VL) was tested by routine assays following manufacturer's instructions. RT, PR and IN genes were sequenced with the ANRS consensus techniques.

Nested PCRs with patient virus IN-specific primers and probes were developed to detect the deleted variant from plasma, blood lymphocytes, rectal biopsies, and sperm.

## Results

VL remained undetectable for more than two years under therapy, excepted for 1 observed blip. Thereafter HIV RNA increased slightly but persistently, fluctuating from 56 to 466 copies/ml during more than five years. By population sequencing a 38 nucleotides deletion was observed in the IN C-terminal domain (CTD) encoding sequence (residues 215 to 227 of the WT IN).

In plasma, the variant progressively emerged during therapy-induced virosuppression, HIV RNA being undetectable by routine viral load assay, and then persisted during detectable viremia. The WT IN, not detected by bulk sequencing, was present but at stable low level.

HIV DNA and RNA with WT IN were amplified from each cell extract. Detection of the deleted IN was a very rare event in blood and rectal cells.

## Conclusions

Although the reservoir of this virus is yet unknown it is likely in a tissue compartment and not predominantly in cells migrating through blood.

Questions arising from this case are how and why this certainly defective variant emerged during efficient virosuppression, and how it could be preferentially and stably produced.

As for interpretation of residual viremia found in long-term treated patients with undetectable viral load, virus production can originate from on-going replication, but the cells had to be continuously co-infected with the two viral forms, or synthesis by chronically infected cells. However, the defective virus production at detectable level suggests unusual circumstances.

Anyway, this case raises concern about the possible long term synthesis of defective viruses.

**Figure 1:**